# Locomotion Mode Recognition Algorithm Based on Gaussian Mixture Model Using IMU Sensors

**DOI:** 10.3390/s21082785

**Published:** 2021-04-15

**Authors:** Dongbin Shin, Seungchan Lee, Seunghoon Hwang

**Affiliations:** Department of Mechatronics Engineering, Hanyang University, 55 Hanyangdaehak-ro, Sangnok-gu, Ansan-si 15588, Gyeonggi-do, Korea; sdbin225@hanyang.ac.kr (D.S.); thetiru@hanyang.ac.kr (S.L.)

**Keywords:** locomotion mode recognition (LMR), gaussian mixture model (GMM), inertial measurement unit (IMU)

## Abstract

The number of elderly people has increased as life expectancy increases. As muscle strength decreases with aging, it is easy to feel tired while walking, which is an activity of daily living (ADL), or suffer a fall accident. To compensate the walking problems, the terrain environment must be considered, and in this study, we developed the locomotion mode recognition (LMR) algorithm based on the gaussian mixture model (GMM) using inertial measurement unit (IMU) sensors to classify the five terrains (level walking, stair ascent/descent, ramp ascent/descent). In order to meet the walking conditions of the elderly people, the walking speed index from 20 to 89 years old was used, and the beats per minute (BPM) method was adopted considering the speed range for each age groups. The experiment was conducted with the assumption that the healthy people walked according to the BPM rhythm, and to apply the algorithm to the exoskeleton robot later, a full/individual dependent model was used by selecting a data collection method. Regarding the full dependent model as the representative model, the accuracy of classifying the stair terrains and level walking/ramp terrains is BPM 90: 98.74%, 95.78%, BPM 110: 99.33%, 95.75%, and BPM 130: 98.39%, 87.54%, respectively. The consumption times were 14.5, 21.1, and 14 ms according to BPM 90/110/130, respectively. LMR algorithm that satisfies the high classification accuracy according to walking speed has been developed. In the future, the LMR algorithm will be applied to the actual hip exoskeleton robot, and the gait phase estimation algorithm that estimates the user’s gait intention is to be combined. Additionally, when a user wearing a hip exoskeleton robot walks, we will check whether the combined algorithm properly supports the muscle strength.

## 1. Introduction

In contemporary society, the percentage of the elderly population has increased continuously as life expectancy increases [1]. This population phenomenon represents an aging society, and elderly people, because muscle strength decreases with aging, have been restricted in activities of daily living (ADL), such as loss of motor function or a fall accident [2]. The ADL comprises seven types [3], and ambulation is the main means of expressing human mobility and must be considered essential in ADL. To be active indoors or outdoors, it is necessary to consider the terrain environment. The essential terrains to consider are level walking (LW), stair ascent/descent (SA/SD), and ramp ascent/descent (RA/RD) [4].

Recently, fusion sensor type researches using various sensors such as inertial measurement unit (IMU) sensors have been actively conducted in relation to ADL, researches are being conducted to determine the terrains [5,6,7,8,9,10,11,12], which are the walking environments or to determine the gait phase related to human intention [13,14,15]. In this study, a pilot study on hip exoskeleton robots as well as locomotion mode recognition (LMR) algorithm for five terrains is considered, and aims to help walking in ADL. The latest studies about LMR algorithm, for being considered the essential five terrains (LW/SA/SD/RA/RD), are divided into fusion sensor types associated with hardware systems and using only sensor types. Regarding the research of the fusion sensor types with hardware, as a pilot study by Samsung, the knee joint angles were predicted by attaching IMU sensors to the back of the lower trunk, the ankle joint, and using the data of the encoder at hip joints. Using these kinematic data, they developed the LMR algorithm using the radial basis function-support vector machine (RBF-SVM) method [5]. In bio-robotics, the LMR algorithm using a back propagation neural network (BPNN) was developed by attaching IMU sensors on the thighs and using the data of encoder sensors at hip joints [6]. For these cases, they developed an algorithm to recognize five terrains by selecting an appropriate artificial intelligence (AI) technique and adding sensors based on the hardware system.

As representative examples of using only sensors, a study conducted by Chen et al. used two IMU sensors and two foot pressure insoles. The IMU sensors were attached to the thigh, shank, and foot, and the linear discriminant analysis (LDA) method was used [7]. Another study conducted by Shahmoradi et al. used a fuzzy basis function and hidden Markov model (HMM) by attaching three IMU sensors and foot pressure insoles to single leg [8]. Additionally, they developed LMR algorithm using various machine learning techniques by attaching seven IMU sensors to the torso, thigh, shank, and foot in the form of a full body. The performance of each was compared [9]. F Sherratt et al. used six IMU sensors attached to the chest, hips, and ankles and determined the five terrains using artificial intelligence technology, long-short term memory (LSTM) [10]. As an example of using the minimum number of sensors, an experiment was conducted on a patient wearing a transtibial prosthesis on one leg, and only one IMU sensor was attached to a toe or heel on a transtibial prosthesis or a healthy leg on the other side. The LMR algorithm was developed using the terrain geometry-based locomotion mode identification system method [11]. Y Han et al. attached one IMU sensor below the knee joint and used a decision tree structure based on using an improved backpropagation neural network (IBPNN-DTS) to classify seven terrains (LW, SA, SD, RA, RD, sitting, standing) [12]. As such, research cases using various machine learning techniques were being actively conducted according to the type or number of sensors used.

Most studies that work with hardware systems were used on the treadmill in indoor environments. There are many cases in which the slope and walking speed were limited. In the case of using only sensors, the experiment was conducted using the user’s usual walking speed (i.e., the optimized walking speed) in outdoor environments since there is no device that adjust the user’s walking speed like the treadmill method. Since the problem that walking speed is different for each age group or user is not considered, if the LMR algorithm previously developed in ADL is applied, problems such as poor classification accuracy may occur. A walking speed environment that considers various age groups is required, and it is necessary to develop an LMR algorithm that can be used not only in indoor environments but also in outdoors. Additionally, it can process data from sensors in real time, and machine learning techniques with high accuracy and high computation speed are required.

To meet these needs, various walking speeds indoor or outdoor environments and real time were considered, and a pilot study was conducted to be applied to robot systems in the future. We used a data-driven method based on an IMU sensor and not on general modeling. The developed LMR algorithm classifies five terrains by dividing the Gaussian mixture model (GMM), a machine learning technique, into two layers. A detailed description of the technology will be discussed in the next chapter.

## 2. Methods

### 2.1. Sensor Systems

**Figure 1:**
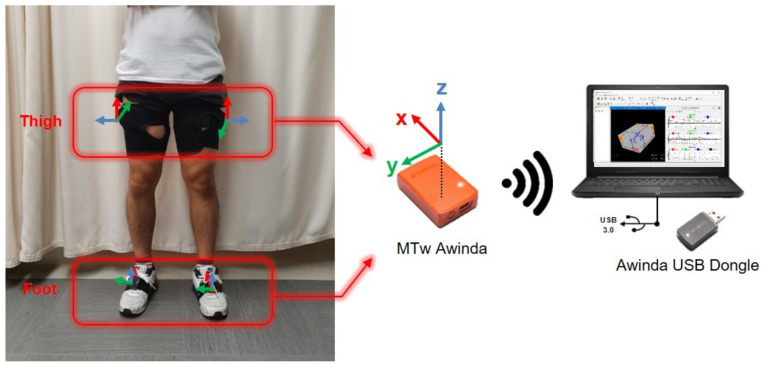
IMU sensors mounted on the lower extremity. 4 IMUs attached to the thigh and foot, respectively. The MTw Awinda sensors communicate with the computer using Awinda USB Dongle.

According to gait analysis, regarding the terrain, level walking and stair terrains are affected by the hip joint angles, while level walking and ramp terrains are affected by the ankle joint angles [16]. The experiment was conducted assuming the result. IMU sensors (Mtw Awinda, Xsens, Enschede, The Netherlands) were attached on the thighs and feet (Figure 1). The number of sensors was four. The data of the sensors were collected on sagittal plane. The data of an accelerometer and gyroscope were collected on the thigh, and the data of a pitch angle were collected on the foot. To collect the data of IMU sensors, the MT manager 4.6 tool provided by Xsens was used, and through the Awinda USB Dongle, the data of IMU sensors were collected. The sampling rate of the data was 100 Hz.

### 2.2. Experimental Protocol

**Figure 2:**
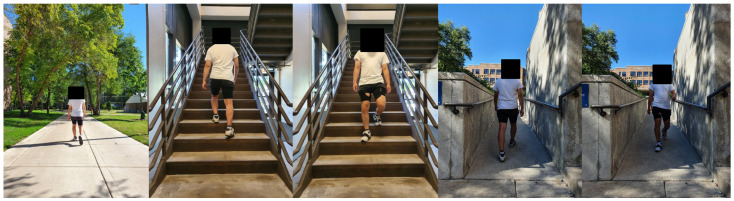
A participant walked the various terrain at the gait speeds corresponding to each BPM 90/110/130 on the over-ground. The terrain was sequentially level walking, stair ascent, stair descent, ramp ascent, and ramp descent (left to right direction).

Unlike the universal method of using the treadmill, each subject walks with a different walking speed on the over-ground. To remove this variable, the beats-per-minute (BPM) method was adopted [17]. The application metronome (Metronome Beats, Stonekick, London, UK) was used. The BPM was divided into three types of BPM 90/110/130. This was divided into walking speed 1.03/1.34/1.57 m/s [18]. In the section of the set walking speed, the index for the walking speed from 20 to 89 years old was used, and the speed range for all age groups was considered [19]. The terrain was divided into five. types (Level walking/stair ascent/stair descent/ramp ascent/ramp descent) (Figure 2). On the level walking, the 6.4 m section was traveled five times, and on the stairs, a total of 14 steps, 1.33 m in length, 0.3 m in width, 0.18 m in height, traveled 10 times. The ramp was a wheelchair slope used in outdoor terrain, and the slope was 5.9 degrees, and the 6.71 m section was rounded 10 times [20]. This study was a pilot study of an exoskeleton robot, and the experiment was conducted with a total of four healthy people (Age: 29.75 ± 3.96, Height: 168.75 ± 6.02 cm, Weight: 66.5 ± 5.59 kg) (Table 1).

The following experimental conditions were defined for the smooth progress of the experiment.

- People walked correctly according to BPM rhythms on the over-ground.
- These were the data of the walking state without obstacles.

## 3. Locomotion Mode Recognition (LMR) Algorithm

### 3.1. Pre-Processing

**Figure 3:**
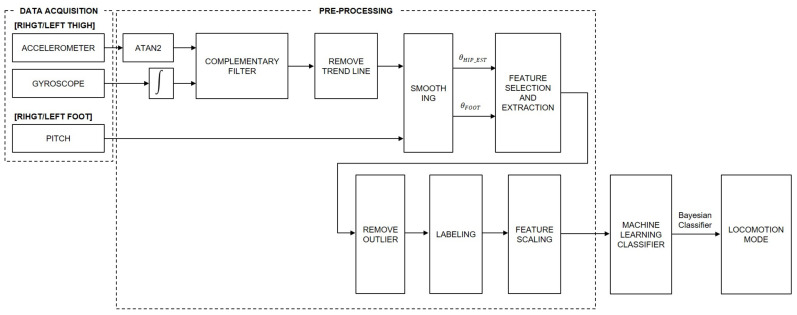
Overview diagram of data analysis. The process comprises the data acquisition, pre-processing, machine-learning classifier, and locomotion mode. Additionally, the pre-processing comprises the estimated hip angle problem, feature selection and extraction, labeling, and the feature scaling.

The pre-processing process was largely divided into data conversion, feature selection and extraction, labeling, and feature scaling (Figure 3). The following process was used in MATLAB R2019b (MathWorks, Natick, MA, USA). In this study, the model for judging terrain was tackled in two ways. The first model was a full-dependent model that collected all the subject data and comprised the data with common principal components. The median method of the isoutlier function in MATLAB was used, and the threshold was applied as 1. Conversely, the second model was an individual-dependent model, which is a model comprising principal components by separately collecting data on individual subjects. In this model method, the threshold of the median method was set to two.

In the estimated angle method using an accelerometer or gyroscope, as the number of IMU sensors used increases, the amount of information in the data increases, inducing an offset phenomenon in the numerical calculation process [21]. To solve the problem, the process of data conversion adopted a method that simultaneously considers the IMU sensor data (accelerometer, gyroscope) of the thigh, and then the estimated angle was converted using the Equations (1) and (2). To reduce the error in posture estimation in static and dynamic conditions through the complementary filter method in Equation (3), a low-pass filter was applied to the accelerometer, and a high-pass filter was applied to the gyroscope [21,22,23]. The trend line was removed to eliminate the drift of the converted angle data [24], and the second-order regression loess and movemean were applied to the estimated hip angle and foot angle, respectively, to remove noise.

(1) $$\theta_{Acc}=atan2\left(\frac{g_{y}}{g_{x}}\right)$$

(2) $$\theta_{Gyro}=\int \omega_{thigh}dt$$

(3) $$\theta_{HipEst}=\alpha \theta_{Gyro}+\left(1-\alpha \right)\theta_{Acc}$$

where $\theta_{Acc}$ is an accelerometer angle. $g_{x}$ and $g_{y}$ are the x and y axis of an accelerometer, respectively. $\theta_{Gyro}$ is a gyroscope angle and the angular velocity measured on the thigh $\omega_{thigh}$ is integrated. $\theta_{HipEst}$ is an estimated hip angle, and the weight factor α=0.99 applies to the filter.

**Figure 4:**
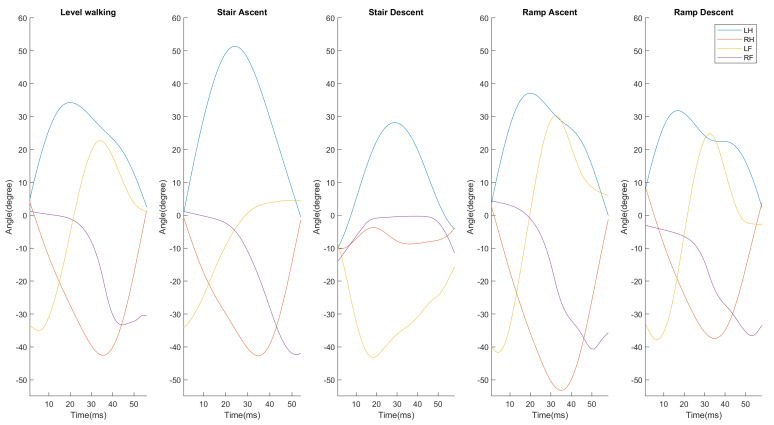
Estimated hip angle and foot pitch angle data for five terrains (LW/SA/SD/RA/RD) for principal component analysis.

The converted data needed to be analyzed using machine learning methods, and the axis was set with data comprising the principal components (PCs) of the data [25]. In the process of feature selection and extraction, the estimated hip angle and foot pitch angle corresponding to the hip cross-point (HCP) to HCP for each terrain to obtain the principal components were used as shown in Figure 4. Classification was possible for LW, SA and SD through the difference between the maximum flexion angle ($\theta_{MaxHip}$) of the hip joint and the extension angle ($\theta_{OppHip}$) of the opposite hip joint (PC1) and the positive value of the foot pitch angle (PC2). However, it was difficult to classify the LW, RA and RD terrains that have the similar gait patterns using the above PCs considered. Therefore the PC was additionally considered, In the single support period, it was confirmed that the corresponding terrains could be classified according to the point where the foot pitch angle was parallel to each other (PC3). The PCs were obtained using Equations (4)–(6).

(4) $$PC1=\theta_{MaxHip}-\theta_{OppHip}$$

(5) $$PC2=\int_{HCP1}^{HCP2}\theta_{LFoot}dt+\int_{HCP1}^{HCP2}\theta_{RFoot}dt\;\left(subject\;to\;\theta_{LFoot}\;and\;\theta_{RFoot}>0\right)$$

(6) $$PC3=\theta_{LFoot}-\theta_{RFoot}\approx 0$$

where PC1 is the amplitude value that is a difference angle between $\theta_{MaxHip}$ and $\theta_{OppHip}$ of the hip joint, PC2 has a positive foot pitch angle and is a value obtained by integrating the $\theta_{LFoot}$ and $\theta_{RFoot}$ between the previous HCP (HCP1) and the next HCP (HCP2). PC3 are the points where $\theta_{LFoot}$ and $\theta_{RFoot}$ were parallel to each other. PC1 and PC3 were used as an important index to determine the slope of the level walking and ramp terrains. To remove the outliers of the PCs, we defined the constraints as less than two median absolute deviation from the median. The labeling process was performed to mark the PCs corresponding to the terrain. Labeling is shown in Table 2 below.

### 3.2. Machine-Learning Classifier

#### 3.2.1. Gaussian Mixture Model (GMM)

The recently developed LMR algorithm uses a machine-learning method that is suitable for preference, depending on the hardware system and data used. To use the algorithm in real time, we needed to consider the machine-learning method for satisfying the high classification accuracy and fast detection of the data. In this study, we analyzed the data using a scatter plot with PCs and confirmed the data, which took the form of a cluster according to the terrain. The GMM, which is unsupervised learning, was used among machine-learning methods to classify clustered data using terrain. Unlike supervised learning, the method classifies data based on probabilistic inference and is a clustering algorithm in which several Gaussian distributions are mixed [26]. As a basic assumption of the mixture model, the probabilistic density function (PDF) for the given data $x_{j},\;j=1,\ldots ,N$ is expressed as a weighted linear sum of the unknown distribution set in Equation (7) [27].

(7) $$f\left(x_{j},\Theta \right)=\Sigma_{k=1}^{K}c_{k}f_{k}\left(x_{j},\Theta_{k}\right)\;\left(subject\;to\;0\leq c_{k}\leq 1,\;\Sigma_{k=1}^{K}c_{k}=1\right)$$

where f(·) is the measure PDF, $f_{k}\left(\cdot \right)$ is the PDF of the mixture *j*, and *k* is the total number of mixtures. Each PDF is weighted by $c_{k}$ and represents the probability of being selected for the *k*th Gaussian distribution as the initial distribution coefficient. $\Theta_{k}$ is an unknown parameter and includes all parameters of the distribution.

For GMM, it includes the two parameters for an unknown parameter $\Theta_{k}:$ mean $\mu_{k}$ and variance $\Sigma_{k}$. $f_{k}\left(\cdot \right)$ is expressed as a conditional probability $N_{k}\left(x_{j}|\mu_{k},\Sigma_{k}\right)$ by central limit theorem, Equations (8) and (9) [28].

In this study, a *d*-dimensional multivariate normal distribution was used, and it was expressed as follows:

(8) $$f\left(x_{k},\Theta \right)=\Sigma_{k=1}^{K}c_{k}N_{k}\left(x_{j}|\mu_{k},\Sigma_{k}\right)$$

(9) $$N_{k}\left(x_{j}|\mu_{k},\Sigma_{k}\right)=\frac{1}{\sqrt{{|\Sigma |\left(2\pi \right)}^{d}}}exp\left(\frac{-1}{2}\left(x-\mu \right)\Sigma^{-1}{\left(x-\mu \right)}^{{}^{\prime }}\right)$$

where μ is the mean and Σ is the variance. The exponent is positive and quadratic. This value is known as Mahalanobis distance. This is a distance normalized by covariance.

#### 3.2.2. Expectation-Maximization (EM) Algorithm

EM is an algorithm that estimates the parameters c,μ,Σ, which make up the GMM for a given data $X=\{x_{1},\ldots ,x_{N}\}$. First, starting from the assumption to estimating the initial parameters Θ, it can be expressed as:

(10) $$\gamma_{p}\left(X,\Theta \right)=\frac{c_{p}f_{p}\left(X,\Theta_{p}\right)}{f(X,\Theta )}=\frac{c_{p}N_{p}\left(X|\mu_{p},\Sigma_{p}\right)}{\Sigma_{k=1}^{K}c_{k}N_{k}\left(X|\mu_{k},\Sigma_{k}\right)}$$

which is the posterior probability of component membership of X in the *p*th distribution. For GMM, the E-step algorithm is expressed as an Equation (10). This expression can be expressed in the form of a normal distribution, and it means the probability that the given data X belongs to the probability density function with parameter $\mu_{p}$, and $\Sigma_{p}$ considers the initial distribution cp ratio within the total probability distribution function. The values of the initial distributions were set as $c_{p}=\left\{\frac{1}{3},\frac{1}{3},\frac{1}{3}\right\}$.

The log-likelihood L(X;Θ) is defined by Equation (11) in the M-step.

(11) $$L\left(X;\Theta \right)=\ln p(X|c,\mu ,\Sigma )=\ln\left\{\prod_{n=1}^{N}p\left(x_{n}|c,\mu ,\Sigma \right)\right\}=\Sigma_{n=1}^{N}\ln\left\{\Sigma_{k=1}^{K}c_{k}N\left(x_{n}|\mu_{k},\Sigma_{k}\right)\right\}$$

Log-likelihood is the same principle as likelihood, and log is used for computational convenience. Likelihood is used in Bayesian theory and refers to the probability of the given data being present in a particular model. Now, if all *X* are independent and have the same probability distribution, the probability density function in the log function can be expressed in product form, Equation (11). Finding the maximizing $c_{k},\mu_{k},\Sigma_{k}$ parameters using the characteristics of monotonic increase can be expressed in the same meaning as the GMM equation. To maximize the log-likelihood, the $c_{k},\mu_{k},\Sigma_{k}$ parameters were partially differentiated over L(X;Θ) [27]. The estimation process of the $\mu_{k},\Sigma_{k},c_{k}$ parameters is shown as follows:

(12) $$\mu_{k}=\frac{\Sigma_{n=1}^{N}\gamma_{k}\left(z_{n}\right)x_{n}}{\Sigma_{n=1}^{N}\gamma_{k}\left(z_{n}\right)}$$

(13) $$\Sigma_{k}=\frac{\Sigma_{n=1}^{N}\gamma_{k}\left(z_{n}\right)(x_{n}-\mu_{k}){\left(x_{n}-\mu_{k}\right)}^{T}}{\Sigma_{n=1}^{N}\gamma_{k}\left(z_{n}\right)}$$

(14) $$c_{k}=\frac{1}{N}\Sigma_{n=1}^{N}\gamma_{k}\left(z_{n}\right)$$

The EM algorithm for GMM calculates an initial $\gamma_{p}\left(X\right)$ for all data X and Gaussian distribution in E-step. In the M-step, a certain number of times is repeated until the parameters for all Gaussian distributions converge to maximum through Equations (12)–(14).

### 3.3. Locomotion Mode Classifier

In this section, the Gaussian distribution of the terrain is calculated for the given data X. In connection with EM algorithm for GMM, the maximized parameters of the probability density function according to *k* terrains are derived, and the probability value $\gamma_{k}\left(z_{n}\right)\in \left\{0,1\right\}$ is calculated using the Bayesian classifier. Among them, the Gaussian distribution with the highest probability value was selected, and the corresponding terrain was classified, Equation (15).

(15) $$y=\arg\max_{k}\gamma_{k}\left(z_{n}\right)$$

### 3.4. Classification Strategy for GMM Algorithm

**Figure 5:**
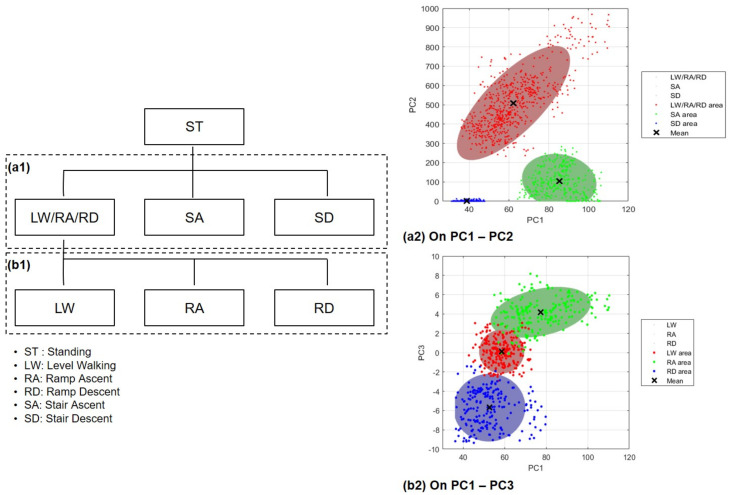
A classification strategy for gaussian mixture model (GMM) algorithm. The strategy comprises two layers. (**a1**) The first layer classifies the stair terrain (i.e., 1st recognizer). In this layer, the LW/RA/RD terrain is classified into the same class. (**a2**) Representative figure of the first layer. The class is classified as follows: (LW, RA, RD-1/SA-2/SD-3) (**b1**) The second layer classifies the level walking and ramp terrain (i.e., 2nd recognizer). (**b2**) Representative figure of the second layer. The class is classified as follows: (LW-1/RA-2/RD-3).

The GMM-based LMR algorithm has a relatively lower probability of success than artificial neural network and recurrent neural network, which are artificial intelligence techniques, but aims to increase the accuracy and does not require much time to classify the data [29]. Therefore, it is possible to use the GMM technique sequentially with the data corresponding to PC1–PC2 and PC1–PC3. The starting point of walking was initially set to standing, to detect which terrain the given data correspond to. In the 1st recognizer, the data on LW, RA and RD are recognized as the same class on the PC1-PC2, and SA, SD are judged as different class. If the given data is recognized as a class of LW, RA and RD, 2nd recognizer is determined which of the three terrains the data corresponds to based on the PC1–PC3 (Figure 5).

### 3.5. Performance Evaluation

Since the two-layer classification methods using GMM sequentially detected the terrain, performance evaluation was conducted by classifying the datasets corresponding to PC1–PC2 and PC1–PC3. The commonly used average classification accuracy method was adopted for the performance evaluation of the algorithm [30], and the confusion matrix, considering the number of labels to be classified, was expressed as follows:

$$C=\left(\begin{array}{ccc} c_{11} & c_{12} & c_{13}\\ c_{21} & c_{22} & c_{23}\\ c_{31} & c_{32} & c_{33} \end{array}\right)$$

where each component is a value obtained by predicted and actual classes, and an accuracy is calculated as (TruePositives+TrueNegatives)/TOTAL×100. To evaluate the reliability of the data, the leave-one-out cross-validation (LOOCV) method was adopted among the validation methods [31]. Through this method, a model is created as many as *N* arrays, and only one sample is used as a test set according to the number of each array. Performance is evaluated through a total of *N* test sets.

## 4. Results

### 4.1. Results of Data Analysis

**Figure 6:**
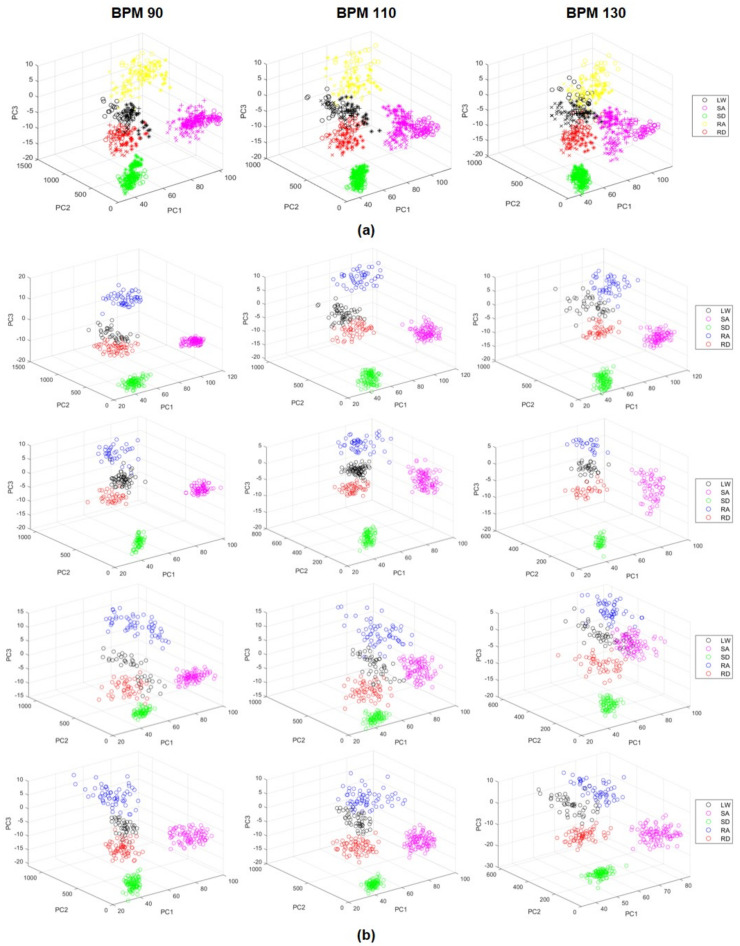
The data of the principal components (PC) on a three-dimensional plane according to beats per minute (BPM) 90/110/130. The terrain is displayed in colors like a legend. (**a**) Full-dependent model case. The subjects are shown as different signs. (**b**) Individual-dependent model case. The data of each subject are arranged in the order of the row.

Figure 6 shows the data on the principal components, and the two models (full/individual dependent model) presented in the study are represented according to BPM 90/110/130. It can be seen that the data on the five terrains comprised clusters within the allocated area, and the degree of clustering varies according to each model or subject. When viewed from PC1–PC2, the data of the stair terrains were less affected by the foot pitch angle and appear to be biased in one direction. In contrast, the data for the level-walking and ramp terrains were clustered in a similar area, but when viewed from PC1–PC3, it showed that the data were clustered by layer. The density distribution of data according to each terrain varies from subject to subject, but as the BPM increased, it shows that the data were scattered throughout. The total data sets of each BPM according to the full-dependent model were 712, 751, and 746, and the individual-dependent models were 1349, 1480, and 1350, respectively.

### 4.2. Results for the Confusion Matrix According to the BPM

The results for the five terrains according to BPM are shown in Table 3. The distribution of the terrain data was dense in a common area for each subject, and as the BPM increases, the classification accuracy for the stair terrain was less affected by the BPM, whereas the level walking and ramp terrains were affected by the BPM. Regarding the full-dependent model, the model used in the confusion matrix was a dependent model using LOOCV. The average accuracy of the test set was 98.74%, 95.78% for BPM 90, 99.33%, 95.75% for BPM 110, and 98.39%, 87.54% for BPM 130 for stair ascent/stair descent and level walking/ramp ascent/ramp descent. The consumption times according to BPM 90/110/130 were 14.5, 21.1, and 14 ms.

For the individual-dependent model, the accuracy of terrain classification was derived for each subject, and the average accuracy of the result was 99.55 ± 0.5%, 95.94 ± 1.11% for BPM 90, 99.58 ± 0.57%, 96.03 ± 0.84% for BPM 110, 98.21 ± 2.73%, and 96.02 ± 1.54% for BPM 130 for stair ascent/stair descent and level walking/ramp ascent/ramp descent. The consumption times according to BPM 90/110/130 are 8 ± 6.68, 7 ± 8.66, and 2.4 ± 1.14 ms. It was confirmed that the accuracy of terrain classification using the individual-dependent model exceeded that of the full-dependent model. Additionally, as the BPM increased, the individual-dependent model showed less error in terrain classification than the full-dependent model.

## 5. Discussion

In most of the existing LMR studies, the studies were conducted in environments selected to optimize walking speed rather than various walking speeds, and the slope was over 10 degrees. The suitable machine-learning method was used depending on the sensor data or the hardware system used. This corresponds to a slope of 18% or more used in the ADL environment. To use it in an actual outdoor or indoor environment, the terrain corresponding to the slope of 10% was considered in order to satisfy the standard maximum ramp slope conditions (8.33–12.5%) [32].

The results, compared with the previous studies, are shown in Table 4, and comparative analysis was performed using a universally used full-dependent model. In the environments of relatively small slope and average walking speed, the classification accuracy of LW and stair terrains is 99.33% and classification accuracy of LW and ramp terrains is 95.75%. The classification accuracy is similar to performance of the existing LMR algorithm, and it is expected that it can be easily used in an environments with an inclination angle of 5.9 degrees or more. In addition, BPM method was used to consider to the walking speed of various people including the elderly people, and the performance of the LMR algorithm for the slow/normal/fast walking speed section and five terrains (LW/SA/SD/RA/RD) was confirmed. In the case of the universally used full-dependent model, it was confirmed that the algorithm performance decreases when the walking speed increases, but when the individual-dependent model was considered, it was confirmed that the classification accuracy was high regardless of the change in walking speed. In this study, as a pilot study of a hip exoskeleton robot, an LMR algorithm was developed that satisfies the walking speed condition (BPM 90) and the actual terrain condition (inclination 10%) for the elderly people. The algorithm uses a GMM, a machine learning method, and it is a model whose computation speed is lighter than that of artificial intelligence methods, and is easy to use in a real-time environment.

The developed LMR algorithm has several limitations. First, in the actual experimental environment, the experiment was conducted for the healthy people and a similar age group, not of the elderly people. However, as the assumption of the experiment, walking was performed according to the BPM rhythm, and the walking speed condition of the elderly people was considered as much as possible. Another limitation is that the number of collected walking data is relatively small. Unlike other research cases, relatively small walking data was used, but as a pilot study, the LMR algorithm was developed based on the trend of the data. Additionally, the terrain classification timing of the algorithm is a steady locomotion mode, not a transition mode in which the terrain changes instantaneously.

As a future study, The LMR algorithm will be applied to the actual hip exoskeleton robot, and the gait phase estimation algorithm that estimates the user’s gait intention is to be combined. Additionally, when a user wearing a hip exoskeleton robot walks, we will check whether the combined algorithm properly supports the muscle strength. Finally, we will recruit the elderly people and use biosensor such as EMG sensors to verify the support validity of the algorithm.

## Figures and Tables

**Table 1:** The characteristics of the tested subjects. Three male subjects and one female subject participated in the experiment.

No.	1	2	3	4
Sex	M	M	M	F
Age	32	31	23	33
Height [cm]	161	173	176	165
Weight [kg]	60	73	71	62

**Table 2:** The labeling table. The terrain was numbered sequentially (level walking (LW)-1, stair ascent (SA)-2, stair descent (SD)-3, ramp ascent (RA)-4, ramp descent (RD)-5).

Terrain	Label
Level Walking (LW)	1
Stair Ascent (SA)	2
Stair Descent (SD)	3
Ramp Ascent (RA)	4
Ramp Descent (RD)	5

**Table 3:** The results of the confusion matrix between the full-dependent model and the individual-dependent model.

No.	Recognizer	BPM 90 (1.03 m/s)	BPM 110 (1.34 m/s)	BPM 130 (1.57 m/s)
**Full-dependent model**				
All	1st	98.75%	99.33%	98.39%
	2nd	95.78%	95.75%	87.54%
Consumption time (ms)		14.5	21.1	14
**Individual-dependent model**				
Subject 1	1st	100%	100%	100%
	2nd	96.5%	96.5%	95.71%
Subject 2	1st	99.37%	99.71%	99.34%
	2nd	97.32%	96.45%	93.75%
Subject 3	1st	98.81%	98.61%	93.51%
	2nd	94.3%	94.58%	98.01%
Subject 4	1st	100%	100%	100%
	2nd	95.65%	96.59%	96.59%
Total	1st	99.55 ± 0.5%	99.58 ± 0.57%	98.21 ± 2.73%
	2nd	95.94 ± 1.11%	96.03 ± 0.84%	96.02 ± 1.54%
Consumption time (ms)		8 ± 6.68	7 ± 8.66	2.4 ± 1.14

1st: classify SA/SD; 2nd: classify LW/RA/RD.

**Table 4:** Locomotion mode recognition comparisons between the proposed method and existing methods.

Reference	Year	Sensor	Placement	No. of Activity	NO. of Subjects	Inclination Angle (Ramp Site)	Classifier	Accuracy	Computation Time (ms)
**[Sensors]**									
Proposed method	2021	4 IMUs	2 thigh, 2 foot	5	4 healthy	$5.9^{\circ }$	GMM	99.33% (SA/SD) 95.75% (LW/RA/RD)	21.1
[7]	2014	2 IMUs, 2 FSR	1 thigh, 1 shank, 2 foot	5	7 healthy	$16.5^{\circ }$	LDA	99.71 ± 0.05%	-
[8]	2017	3 IMUs, 1 FSR	1 thigh, 1 shank, 2 foot	7	4 healthy	$15^{\circ }$	Fuzzy sequential pattern recognition /HMM	95.8% /86.5%	-
[9]	2020	7 IMUs	1 torso, 2 thigh, 2 shank, 2 foot	5	10 healthy	$10^{\circ }$	Gaussian SVM	99.8 ± 0.3%	-
[11]	2020	1 IMU	1 heel	5	3 healthy, 3 amputee	$7^{\circ }<x<15{\;}^{\circ }$	Elliptical boundary	98.5%	-
[12]	2021	1 IMU	1 knee joint	6	6 healthy	$9^{\circ }$	IBPNN- DTS	97.29%	-
[10]	2021	5 IMUs	1 chest, 2 hip joints, 2 ankle joints	5	22 healthy	-	LSTM	Above 95%	-
**[Hip exoskeleton robot + Sensors]**									
[5]	2017	3 IMUs, 2 encoders	1 torso, 2 hip joints, 2 ankle joints	5	5 healthy	Above $10^{\circ }$	RBF-SVM	99.3% (LW/SA/SD) 95.45% (RA/RD)	-
[6]	2020	2 IMUs, 2 encoders	2 thigh, 2 hip joints	6	3 healthy	$10^{\circ }$	BPNN	98.43% (zero-torque) 98.03% (assistive mode)	0.9

## Data Availability

The data presented in this study are available on request from the corresponding author.

## Abbreviations

The following abbreviations are used in this manuscript:

ADL	Activities of Daily Living
LMR	Locomotion Mode Recognition
GMM	Gaussian Mixture Model
BPM	Beats per Minute
PC	Principal Component
LOOCV	Leave-One Out Cross-Validation
